# Long‐Term Multidisciplinary Management of Regional Odontodysplasia: A Case Report

**DOI:** 10.1155/crid/1604573

**Published:** 2025-12-29

**Authors:** Thalassia Niarchou, Katerina Koleventi, Katerina Papadimitriou, Filomeni Anagnostou, Olga-Elpis Kolokitha, Konstantinos Arapostathis, Eleni Kotsiomiti

**Affiliations:** ^1^ Department of Prosthodontics, School of Dentistry, Faculty of Health Sciences, Aristotle University of Thessaloniki, Thessaloniki, Greece, auth.gr; ^2^ Department of Pediatric Dentistry, School of Dentistry, Faculty of Health Sciences, Aristotle University of Thessaloniki, Thessaloniki, Greece, auth.gr; ^3^ 251 Hellenic National Air Force & VA Hospital, Athens, Greece; ^4^ Department of Orthodontics, School of Dentistry, Faculty of Health Sciences, Aristotle University of Thessaloniki, Thessaloniki, Greece, auth.gr

**Keywords:** case report, multidisciplinary, prosthodontic, regional odontodysplasia

## Abstract

Regional odontodysplasia (RO) is a rare developmental anomaly involving both mesodermal and ectodermal dental tissues. It is characterized by hypoplasia and hypomineralization of enamel and dentin affecting a group of neighboring teeth and may involve primary, permanent dentition or both. Wide pulp chambers and thin, poorly defined hard tissue outlines, described as “ghost teeth,” are typical radiographic features. A case of the long‐term management of a young girl suffering from RO in the left maxilla is described in the present report. The patient was 3 years old when first presented in the clinic and was treated for the subsequent 12 years by a multidisciplinary team comprised of a pediatric dentist, orthodontist, and prosthodontist. The report encompasses a detailed description of the clinical and radiographic findings of the patient. It also describes the prosthodontic treatment applied to alleviate the aesthetic handicap brought by the extraction of the affected teeth, as well as the orthodontic interventions to establish the normal eruption and settlement of the remaining dentition. A continuing and steady follow‐up of RO cases is necessary in order to keep pace with the needs arising from the loss of affected teeth and to ensure the normal growth of the oral issues.

## 1. Introduction

Regional odontodysplasia (RO) is a rare, nonhereditary developmental anomaly affecting dental tissues derived from both the mesoderm and ectoderm [1, 2]. The condition was probably first described by Hitchin [3], but the term “odontodysplasia” was introduced by Zegarelli et al. in 1963 [4]. The prefix “regional” was added by Pindborg in 1970 [5, 6]. The prevalence of this condition is not clear, since the current information has been mainly based on case reports; around 180 cases have been reported in the literature [7]. In 2022, Nijakowski et al. published a systematic review, which summarized the existing knowledge on localization, symptomatology, and treatment methods for RO patients, based on published case reports from 28 countries and 180 patients′ data [8]. The authors reported that RO occurs in both deciduous and permanent dentition, regardless of the sex of the patients. The affected teeth were observed more frequently in the maxilla (70.0%), especially on the left side (45.6%). Generalized odontodysplasia was described in only 5.5% of the patients [8].

The diagnosis of RO is based on clinical and radiographic findings. Clinically, the affected teeth have a discolored, hypocalcified, hypoplastic appearance [8, 9] and small size. Delayed or incomplete eruption is a common finding [8, 10]. Pus or fistulas appear near the affected teeth without the presence of decay [11–14]. Considering gender, in female patients, gingival swelling has been observed significantly more often than in males.

On radiographic examination, enamel and dentin are nondistinguishable, giving the teeth a “ghost‐like” appearance. The calcification patterns are disordered, and the pulp chambers are enlarged. According to the systematic review of Nijakowski et al. [8], the radiographs practically always show ghost teeth (100.0%) and poorly developed buds (92.2%) in the affected quadrants, similarly for male and female patients. The modern radiographic techniques, such as cone beam computed tomography and three‐dimensional imaging, have greatly improved the diagnosis of RO and facilitated its treatment [15–18].

The histological characteristics of RO include a loose, inflamed connective tissue with calcified bodies in the dental sac, areas of cellular dentin along with amorphous areas within the affected tooth′s coronal dentin and a gray appearance in stained hematoxylin and eosin‐stained sections [2, 19]. Interlobular dentin regions are common [20], as well as invaginations extending from the enamel surface to the dentin, which permit the entry of bacteria, leading to pulpitis [13].

The etiology of RO is unknown. Various etiological factors have been proposed to explain its pathogenesis, including trauma, infection, blood supply disturbances, medication, radiotherapy, fever, metabolic disorders, and nutritional deficiencies [21]. Heredity does not seem to play a role, as no confirmed inherited cases have been reported [8, 22]. Nonetheless, the condition has been reported in association with certain medical conditions such as vascular nevus, [22–24], epidermal nevus syndrome [25], orbital coloboma [26], and hydrocephalus [27].

The aim of this report was to present the long‐term management of a young girl with RO in the left maxilla. The patient was first examined at the age of 3 years and received care from a multidisciplinary team, including a pediatric dentist, orthodontist, and prosthodontist, for the next 12 years. Following the presentation of the patient′s clinical and radiographic observations, the report details the prosthodontic treatment used to improve the appearance after removing affected teeth, along with orthodontic measures implemented to ensure proper eruption and alignment of the remaining teeth.

## 2. Case Report

A 3‐year‐old girl presented to the Postgraduate Department of Pediatric Dentistry of the Dental School in April 2009. Her mother was 30 years old, and her father was 35 years old; she was the first child, and all members of the family had a clear medical history. The parents′ chief complaint was swelling around unerupted teeth.

**Figure 2:**
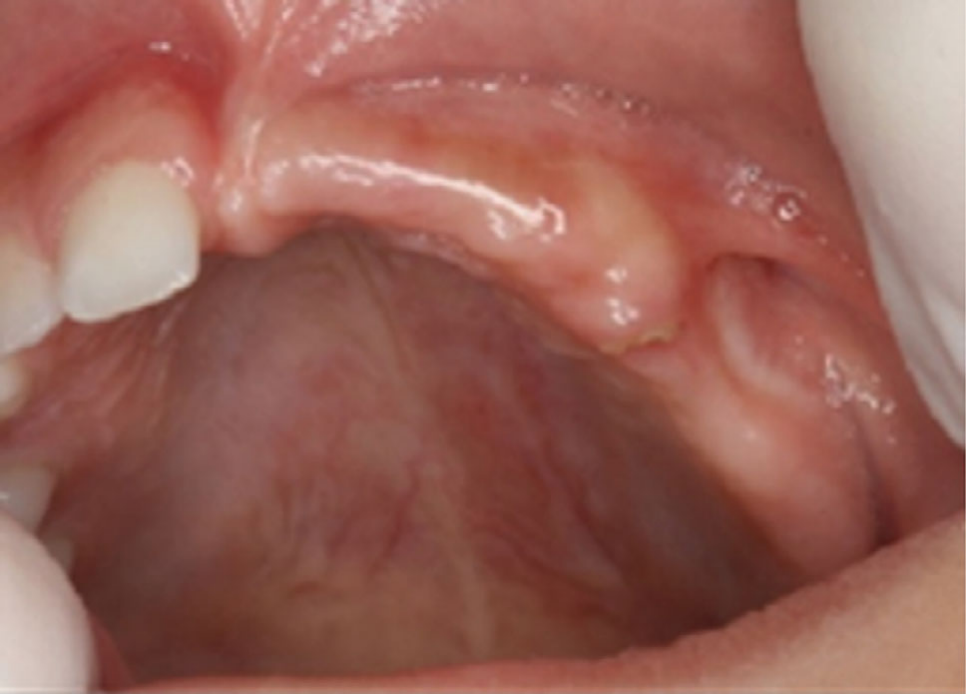
Clinical presentation following the extraction of Teeth #61–#65.

The clinical examination revealed that teeth #61–#64 were discolored, hypocalcified, and hypoplastic. They were of a yellow–brown color, abnormal morphology, and soft upon probing; they appeared not fully erupted and were surrounded by hyperplastic gingival tissues. The crowns of Teeth #61 and #62 were completely worn away, and the crowns of Teeth #63 and #64 were small and conical (Figure 1a). The periapical radiographs of Teeth #61–#65 showed that the enamel and dentin were indistinguishable, exhibiting the typical “ghost‐like” appearance and the pulp chambers appeared enlarged (Figure 1b). Based on the above findings, a diagnosis of RO was made. Due to the condition of the involved teeth, their extraction was advised. The parents gave their written consent for the extractions. The “Ghost Teeth” #61–#64 were extracted in June 2010 under general anesthesia. At that time, Tooth #65, also affected, had erupted and was extracted too (Figure 2).

Figure 1(a) Clinical and (b) radiological appearance of affected teeth. Patient′s age: 3 years old.

**Figure:**
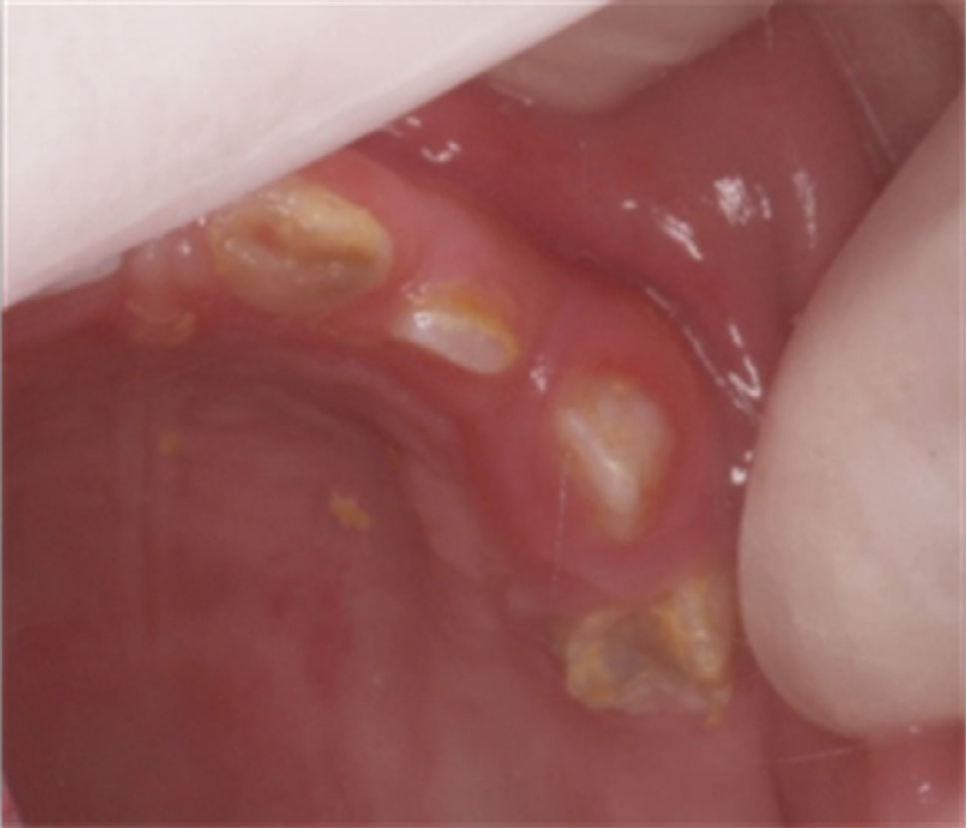
(a)

**Figure:**
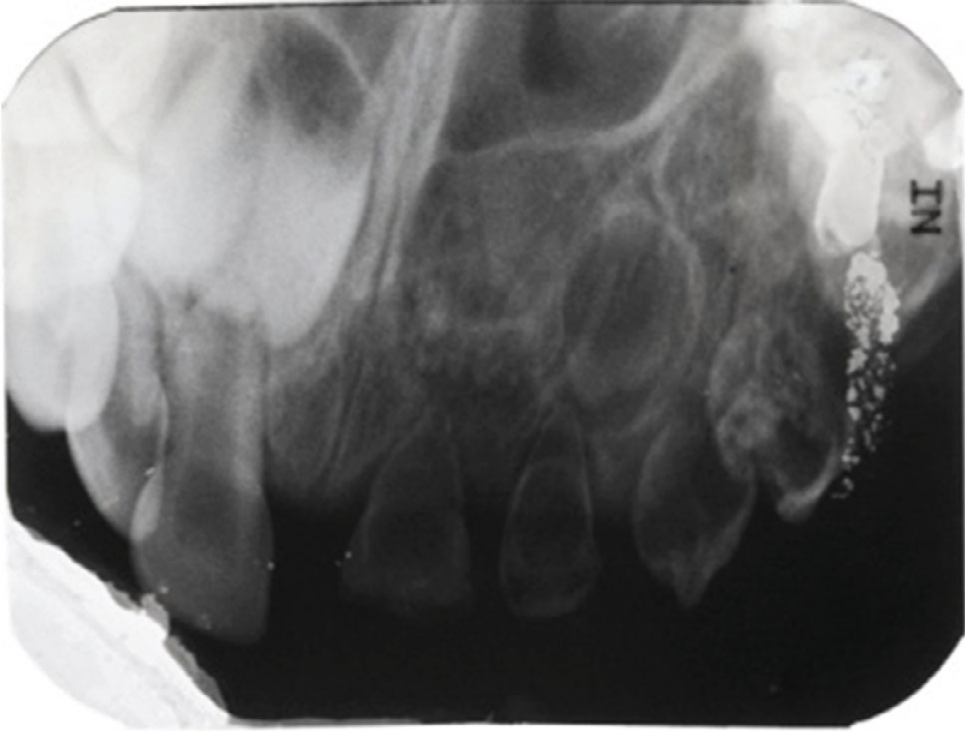
(b)

Consequently, the patient was admitted to the unit for treatment of children and adolescents with multiple dental agenesis of the dental school. The treatment plan was organized by the pediatric dentists, orthodontists, and prosthodontists who are members of the team. It included consistent follow‐up of the patient by all the above disciplines and targeted interventions when needed. The parents were informed accordingly and gave their written consent.

**Figure 3:**
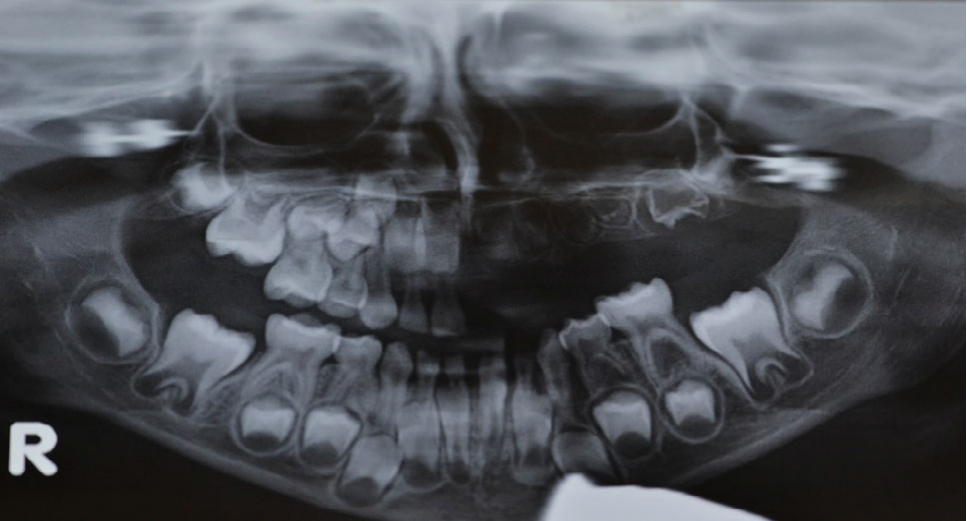
Orthopantomogram of the patient at age 6.

For the next 3 years, the child was placed under pediatric follow‐up every 6 months. In every visit, professional cleaning was performed and behavior management techniques were applied in order to gain the child′s cooperation. At the age of 6 years, a panoramic x‐ray was prescribed and revealed that all permanent teeth of the left maxilla (#21–#26), which had not yet erupted, presented the typical “ghost teeth” appearance. The second molar (#27) had not been formed at that time (Figure 3).

**Figure 5:**
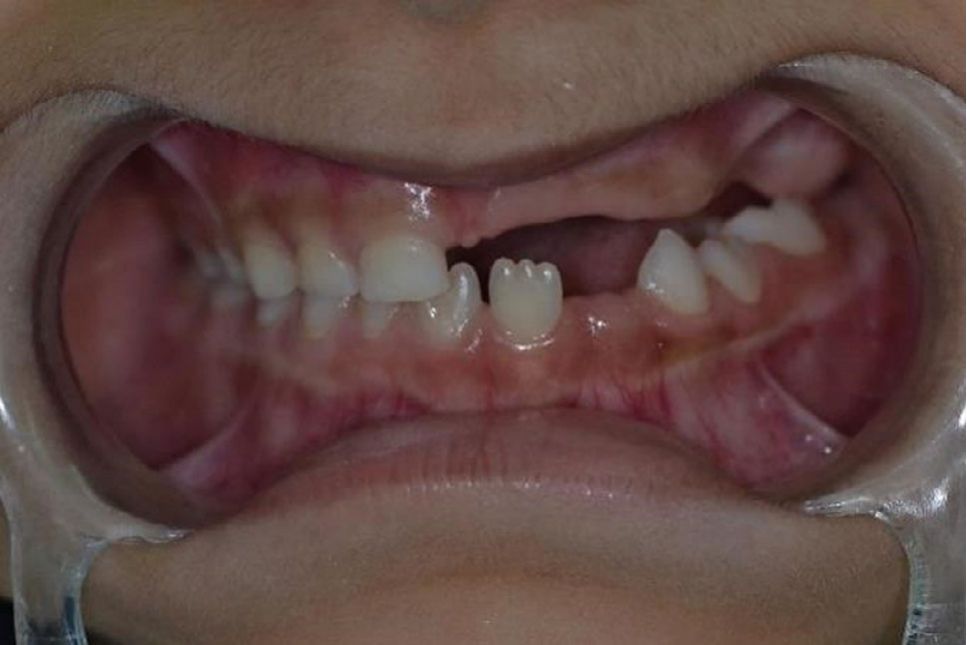
Frontal view of the patient at maximum intercuspation. Age: 7 years old.

At patient′s age of 7 years, the construction of an interim removable partial denture (RPD) was decided. The purpose was to replace the missing teeth, alleviating the aesthetic handicap, and also to assist the oral function and maxillary growth. Preliminary impressions were made using irreversible hydrocolloid material (Kromopan, Lascod). *Τ*he study casts were used for the fabrication of custom acrylic resin trays, which in turn served for the final impressions again of irreversible hydrocolloid (Kromopan, Lascod) (Figure 4). The final casts were articulated at maximum intercuspation, according to the maxillomandibular records. Although the space between the upper edentulous ridge and the occlusal surfaces of the lower teeth was severely restricted (Figure 5), we managed to set up primary acrylic artificial teeth at the patient′s existing occlusal vertical dimension. No modifications of the natural teeth were performed, and the RPD was mucosa‐borne. The retention was supplied by the base adaptation and assisted by two wrought wire clasps, engaging Teeth #51 and #16. The denture was tried, finished, delivered, and modified as needed. Subsequently, the denture was sectioned at the midline, and an expansion screw was added in order to accommodate the maxillary growth (Figure 6). The parents were instructed to activate it in regular intervals. The patient and parents were set into a 3‐month recall program.

Figure 4(a) The custom acrylic resin trays for the construction of the first RPD. (b) Final impression.

**Figure:**
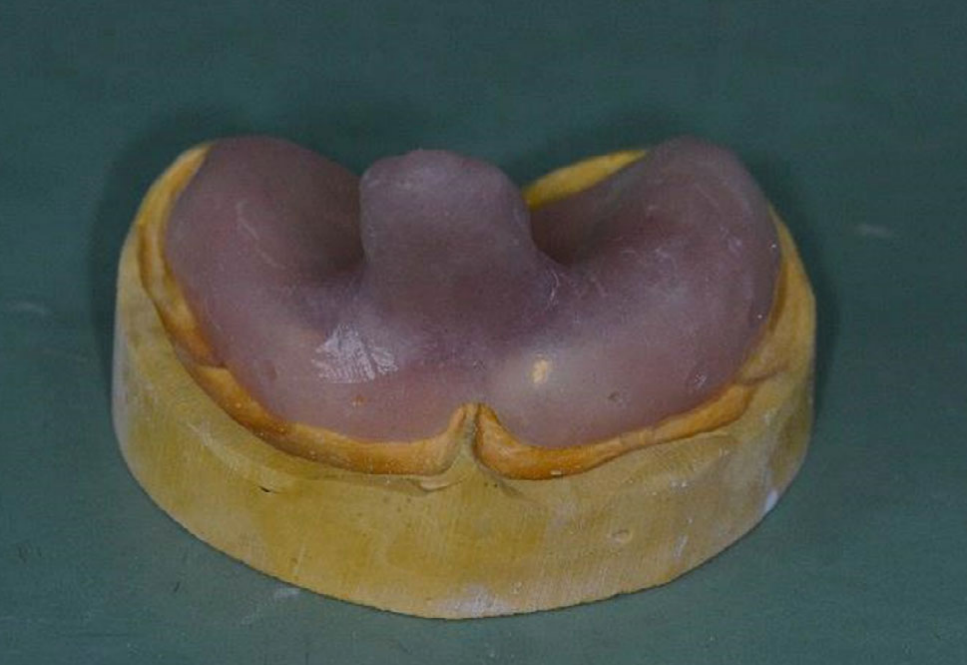
(a)

**Figure:**
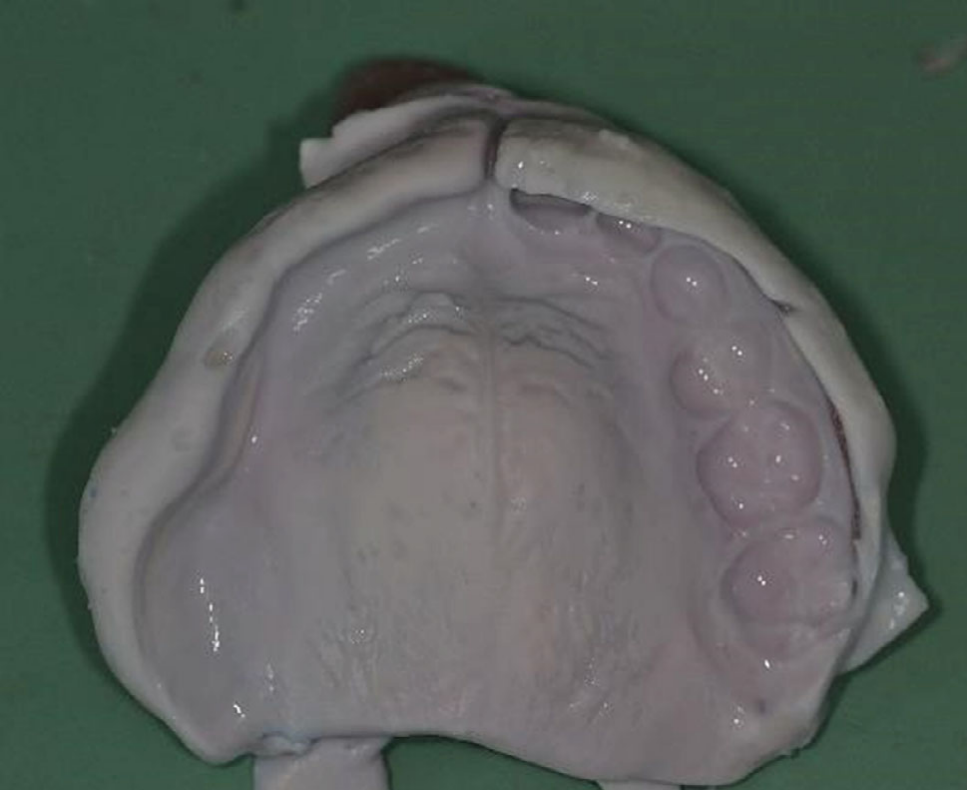
(b)

Figure 6(a) First interim RPD with expansion screw. (b) The RPD in place. Age: 7 years old.

**Figure:**
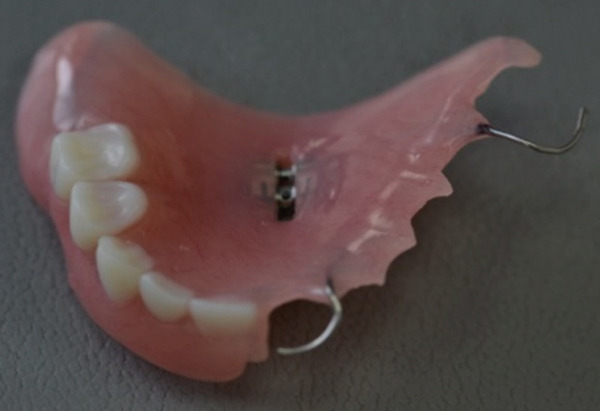
(a)

**Figure:**
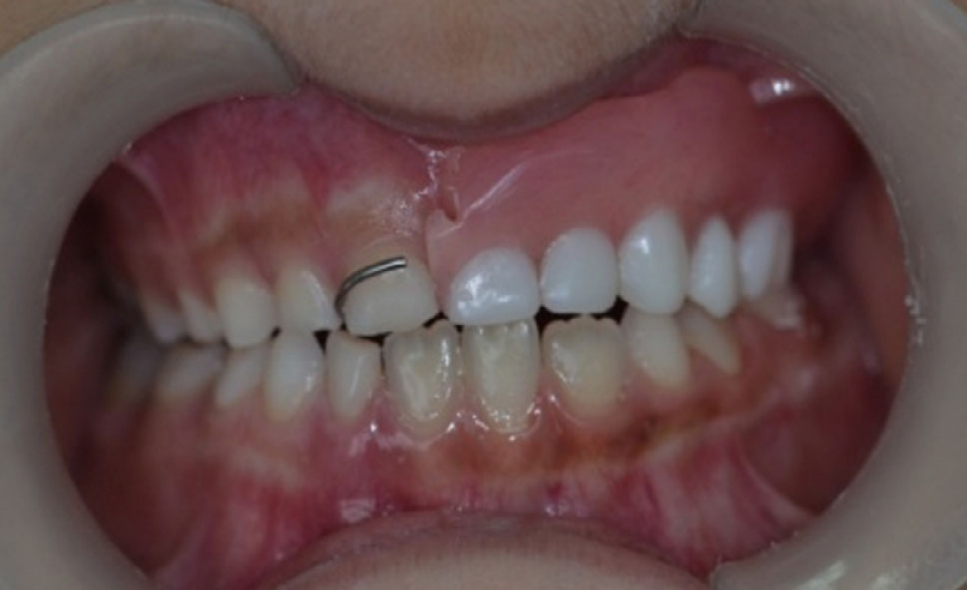
(b)

However, they missed some of the programmed appointments. The patient was again examined 14 months later, at her 9 years, and after a 6‐month period of noncontact. She was not wearing the denture. On examination, it was evident that the denture base had lost its fit. This occurred because the maxillary tuberosity was augmented due to the growth of the sperm of the second molar, which was enclosed under it. A replacement of the denture was decided. However, the patient was rather reluctant about the new denture and not enthusiastic about her appointments. Additionally, the family had to temporarily move out of the city. Therefore, they decided not to continue with the prosthodontic rehabilitation.

The family followed the 6‐month pedodontic prophylaxis recalls for the next years. At the age of 9 and 13 years, extraction was carried out of Teeth #26 and #22, respectively. Following the extraction of Tooth #26, histologic examination revealed the characteristic features of RO (Figure 7). The teeth were extracted immediately after their eruption and under local anesthesia. She also had resin‐modified glass ionomer restorations on #74, #54, and #55. Fissure sealants were placed on two first permanent molars (#16 and #46), while #36 had a composite resin restoration.

Figure 7Histological sections of Tooth #26 after its extraction. (a) Enamel–dentin junction. (b) Disruption of the ameloblast layer. (c) Abnormal dentinogenesis with sparse odontoblasts. (d) Poorly developed cementum and widened pulp spaces. Age: 9 years old.

**Figure:**
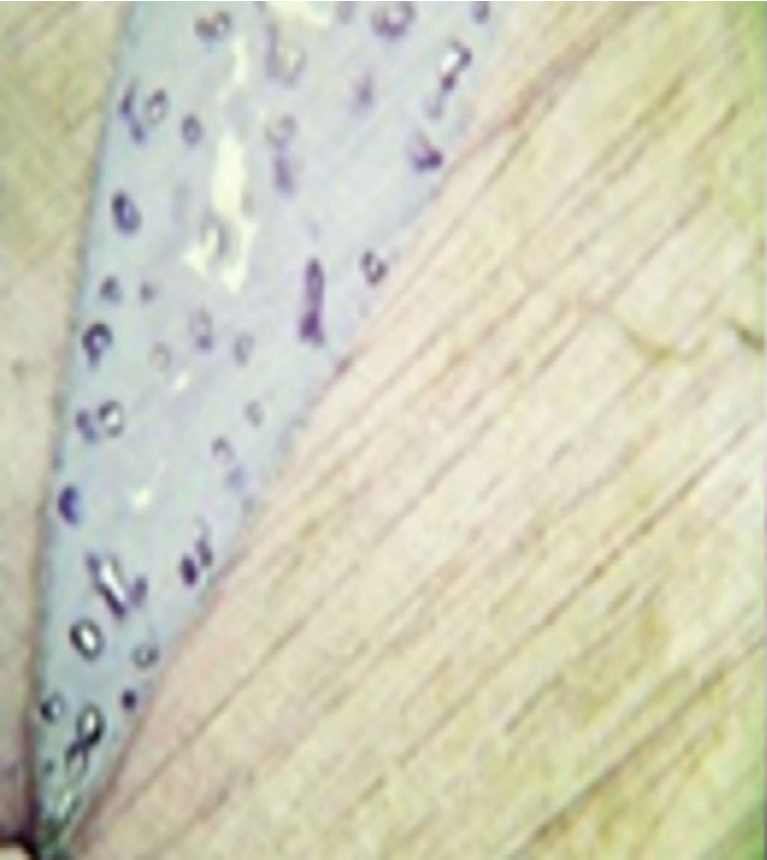
(a)

**Figure:**
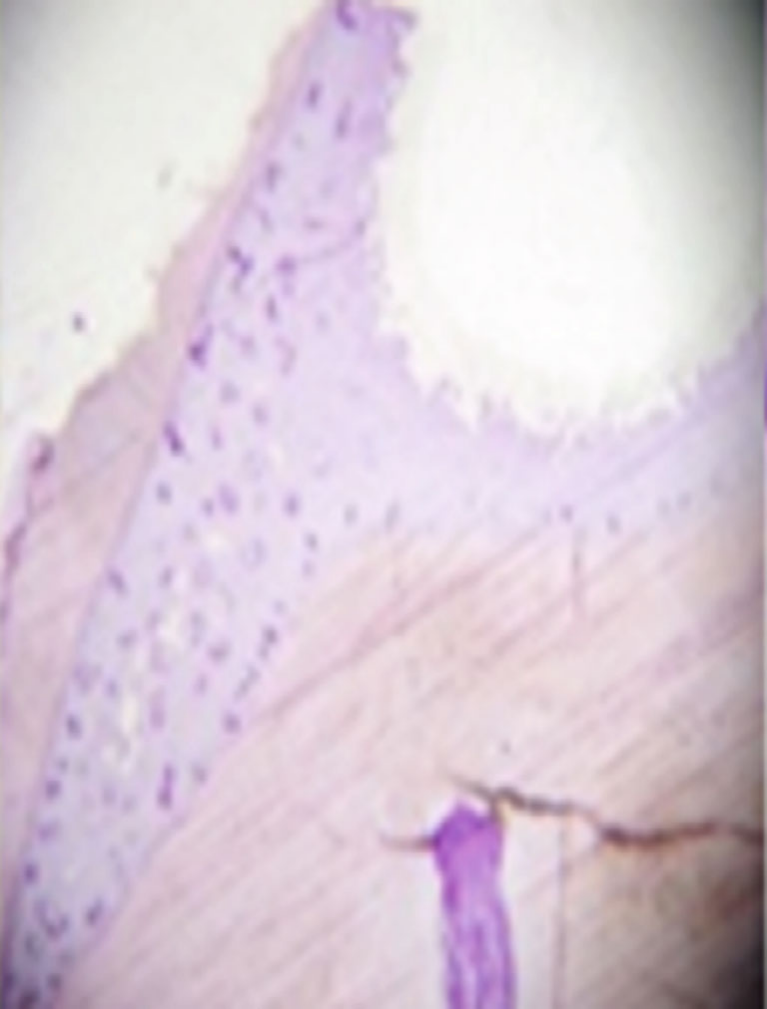
(b)

**Figure:**
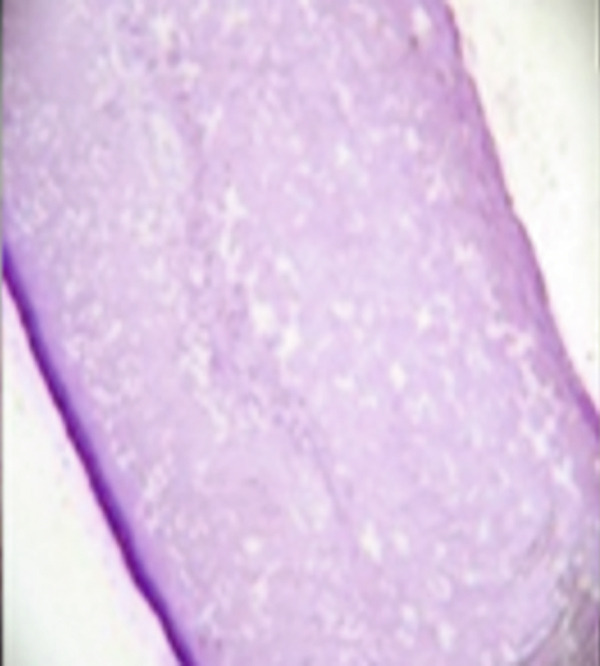
(c)

**Figure:**
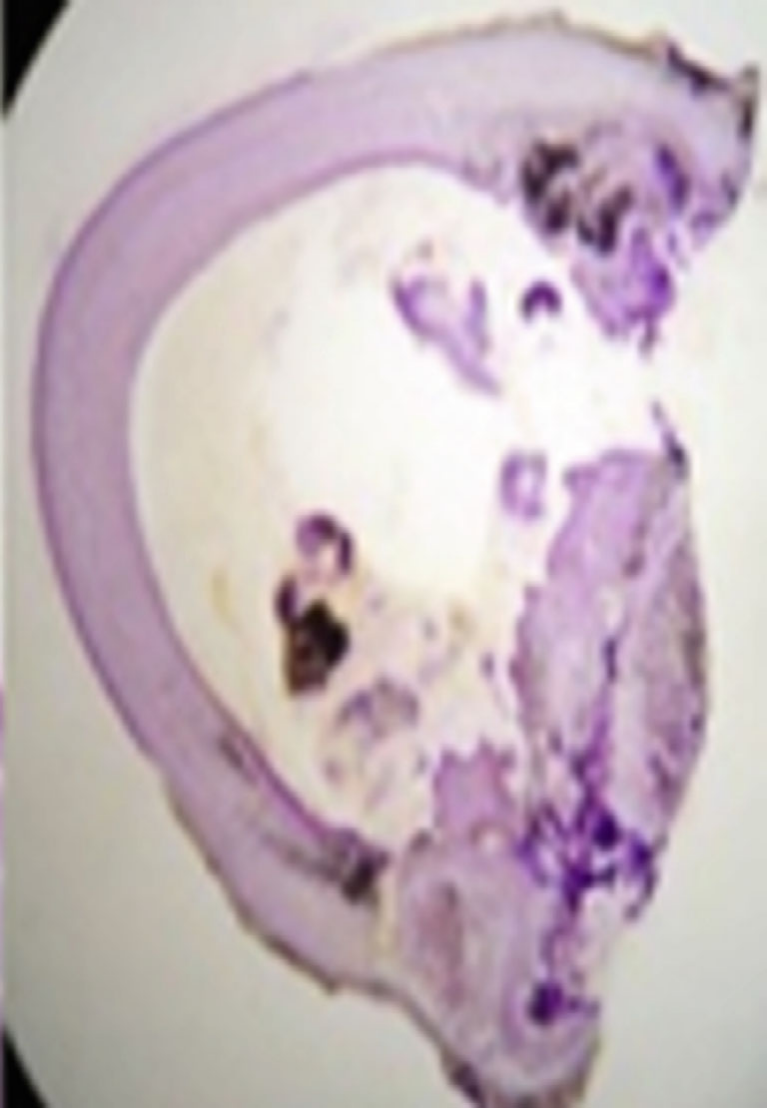
(d)

In 2021, at the age of 15 years, the patient expressed the desire to have a replacement for her missing teeth and actively asked for the construction of a removable device to fill the edentulous area. In view of the second prosthodontic intervention, the multidisciplinary team consulted to update and organize the treatment plan. Panoramic, occlusal, and periapical radiographs were again obtained.

**Figure 8:**
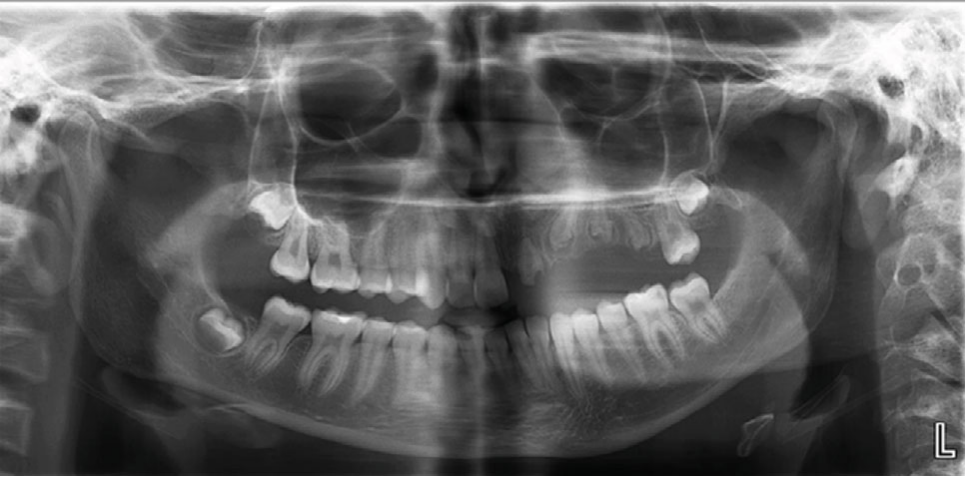
Orthopantomograph of the patient at age 15.

Teeth #27 and #28 were the only unaffected teeth in the second quadrant. Tooth #27 had fully erupted, and #28 was recorded radiographically. Teeth #23, #24, and #25 remained impacted. They presented the typical “ghost‐like” appearance, with an insignificant amount of root formation (Figure 8). The central incisor (#21) had erupted partially. It was yellowish in color, soft in consistency, and had a short unformed root. The unaffected permanent teeth had erupted. Because of the lack of antagonists, the left mandibular incisors (#31 and #32) had extruded. The maxillary left second molar (#27) was also extruded, intercepting the normal eruption of the lower left second molar (#37), which was submerged (Figure 9). On orthodontic examination, the patient had a short facial pattern, with a straight profile of soft tissues and reduced nasolabial angle. She had permanent dentition with Class I malocclusion and Class I skeletal relationships. The patient maintained good oral hygiene, conforming to the instructions given by the pediatric dentists.

Figure 9Clinical presentation of the patient at age 15. (a) Frontal view and (b) occlusal view.

**Figure:**
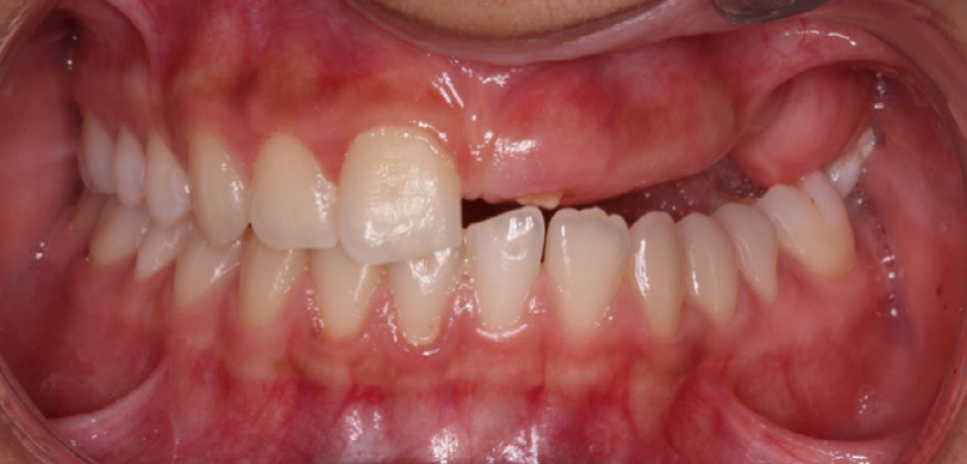
(a)

**Figure:**
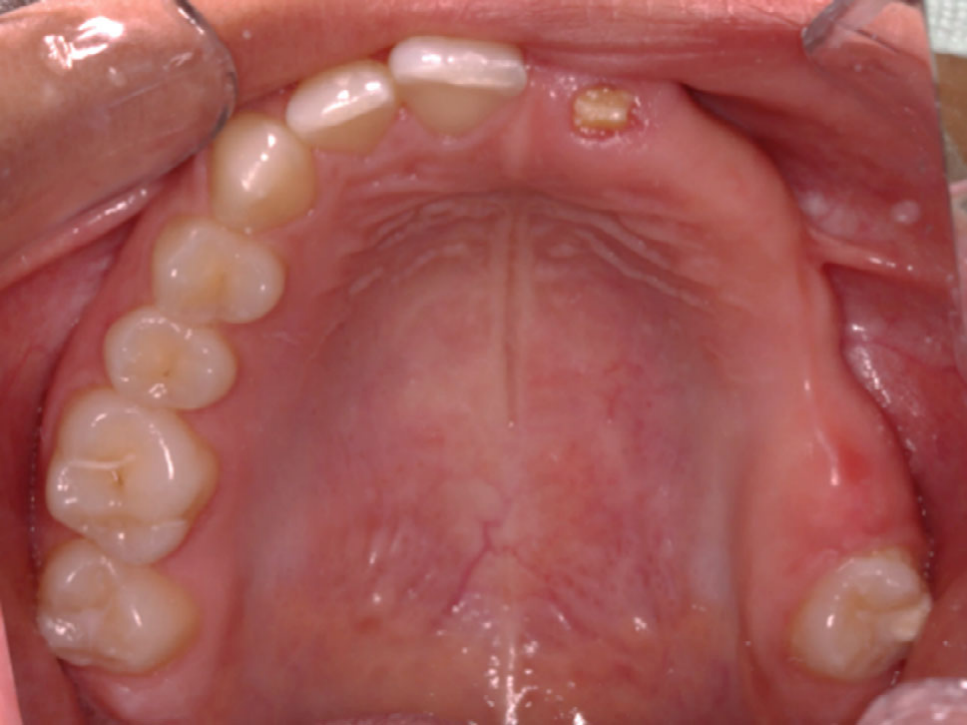
(b)

The multidisciplinary treatment team decided to extract the affected central incisor (#21). The impacted ghost teeth would be preserved to prevent bone atrophy. Orthodontic treatment would precede the prosthodontic management, aiming to level the curve of Spee by relative intrusion of the mandibular incisors and elongation of the premolars. An interim RPD would then be constructed by the prosthodontists.

**Figure 10:**
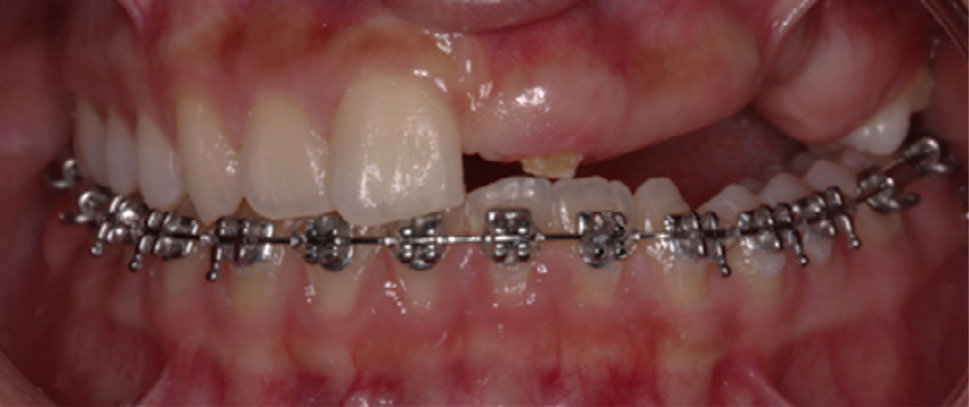
Installment of fixed orthodontic appliances in the mandibular arch. Age: 15 years old.

The orthodontic treatment started with the placement of fixed orthodontic appliances (brackets 018 ^″^ Roth prescription) in the mandibular arch, up to the second molars (Figure 10). Alignment and leveling were accomplished with continuous archwires (0.012 ^″^, 0.014 ^″^, 0.016 ^″^, 0.016^″^ × 0.016^″^, and 0.016^″^ × 0.022^″^ NiTi) at the next appointments. Leveling was achieved by intrusion of the lower incisors and elongation of the lower premolars. At the eighth month of the treatment, rigid stainless steel archwires were placed (0.016^″^ × 0.022^″^ SS) to close some minor spaces, which were created during the treatment. Bends were made in the archwire, for better settling of the teeth, to ensure a stable result after treatment. After 9 months, the fixed orthodontic appliances were removed and a fixer retainer was bonded in the mandibular lingual anterior region (#33–#43).

The extraction of Tooth #21 and the orthodontic treatment effectively increased the prosthodontic space at the edentulous area for the fabrication of the second provisional partial denture. To speed up the construction of the denture, the prosthodontic procedures started immediately after the leveling of the occlusal plane, at 8 months of orthodontic treatment, and proceeded in parallel with the final steps of the latter. Impressions of the maxilla and mandible were made using irreversible hydrocolloid impression material (Kromopan, Lascod) and utilized to construct the working casts.

The remaining teeth supported a stable occlusion and a normal occlusal vertical dimension, which were maintained for the RPD. An interocclusal record at the maximum intercuspation was taken using an acrylic base with a wax rim. Gray wax (Aluwax, Aluwax Dental Product Co., United States) was used to record contacts bilaterally (Figure 11). The casts were mounted on a semiadjustable articulator.

Figure 11Interocclusal record. (a) Frontal view and (b) occlusal view.

**Figure:**
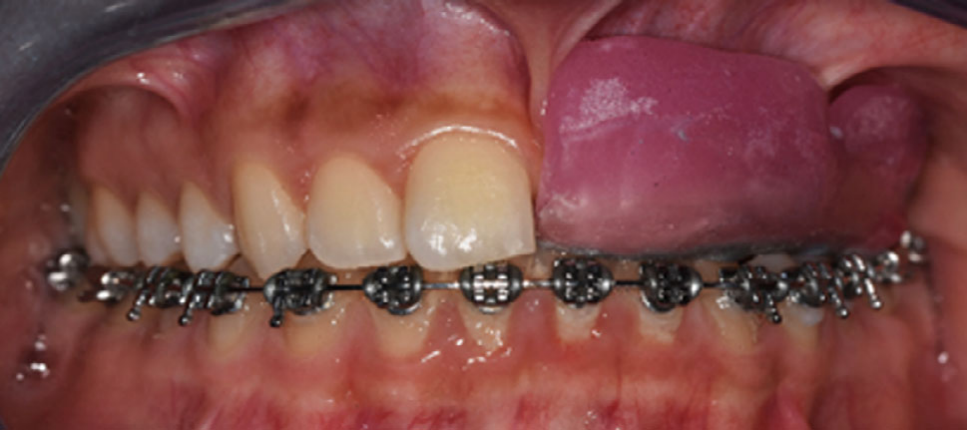
(a)

**Figure:**
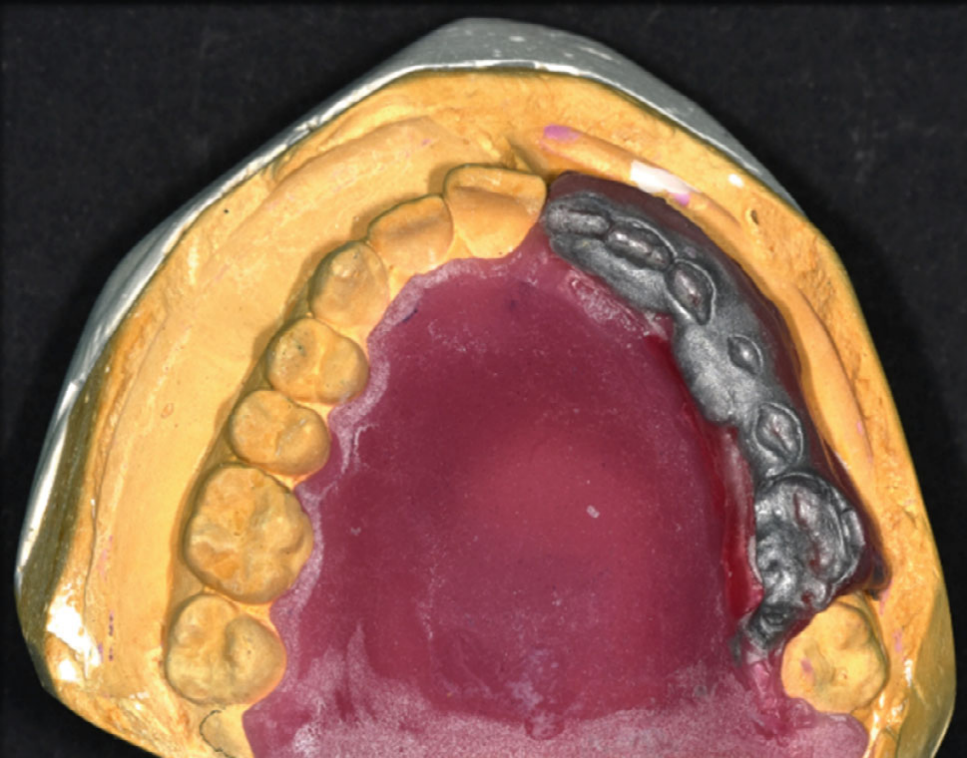
(b)

As aesthetics were of prime importance for the patient, the color of the acrylic teeth was carefully chosen. The teeth were set up, trimmed, and modified where needed, in order to match the appearance of the natural dentition. The denture and teeth set‐up were tried in the mouth, and their position and aesthetics were checked and approved (Figure 12).

Figure 12(a) Acrylic teeth set‐up and (b) denture try‐in.

**Figure:**
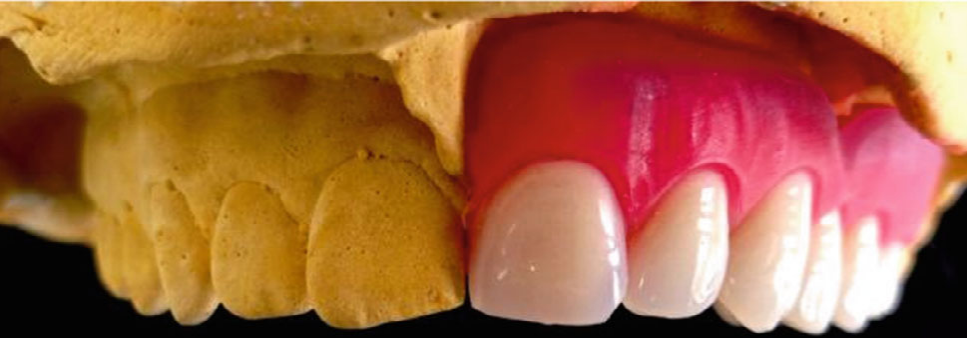
(a)

**Figure:**
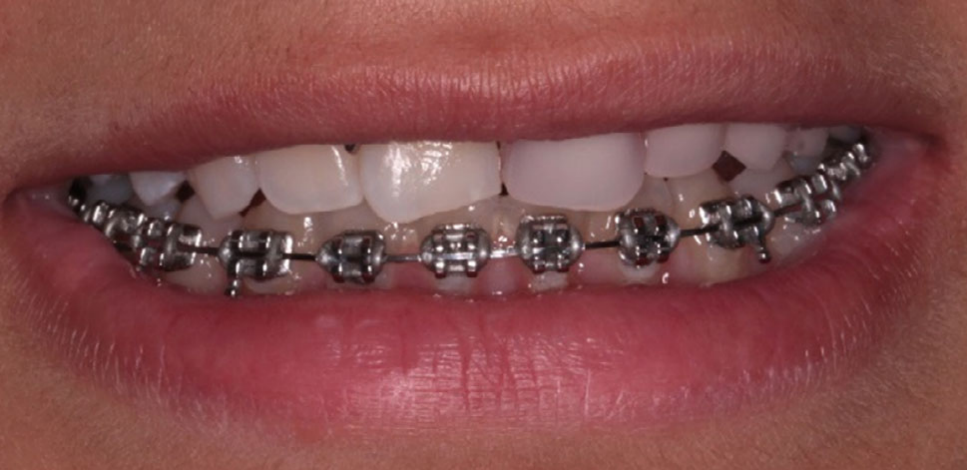
(b)

The second RPD was again mucosa‐borne and was retained by two wire clasps engaging undercuts of the right central incisor (#11) and second molar (#17). The acrylic base covered only the anterior part of the palate, providing maximum comfort for the young patient (Figure 13).

Figure 13The finished second RPD (a) at the day of delivery and (b) at 1‐week follow‐up.

**Figure:**
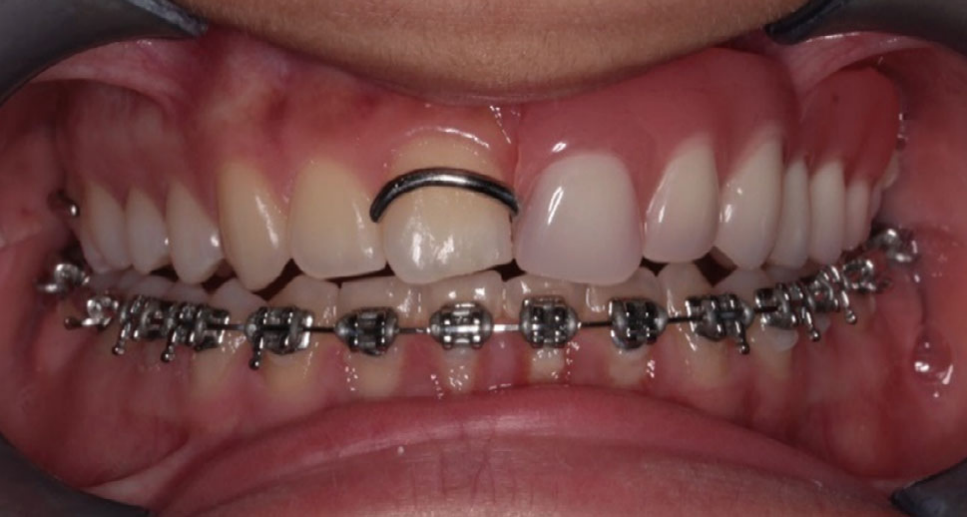
(a)

**Figure:**
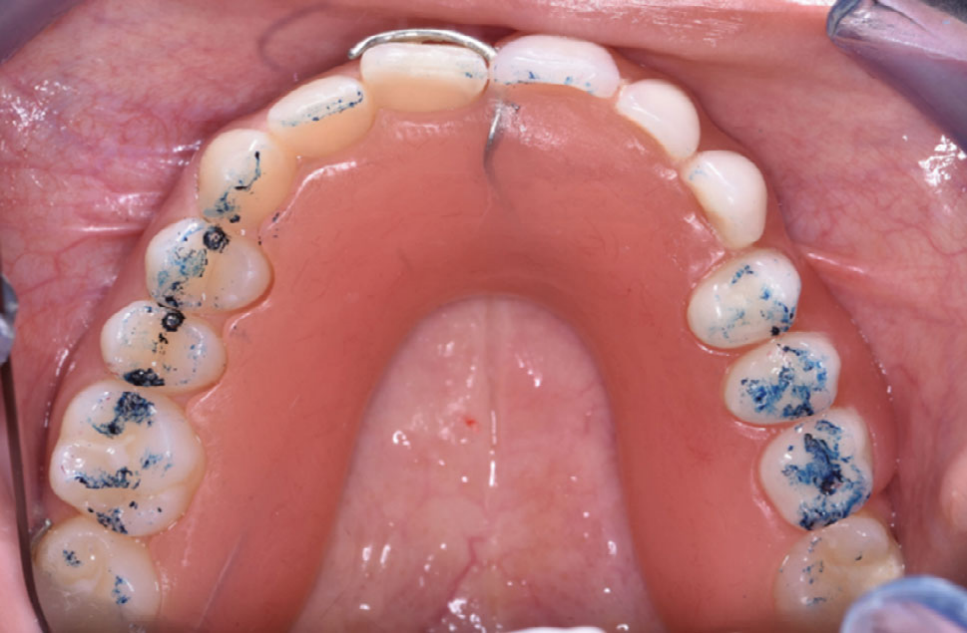
(b)

Upon delivery of the denture, instructions were given for proper use, cleaning, and maintenance. The patient was examined 2 days after the delivery. She was already adjusted to the denture, able to talk, eat, and smile in confidence. As the upper lip did not reveal the frontal clasp while smiling, the aesthetic result was quite satisfying. One week later, an occlusal equilibration took place, at the expense of the acrylic teeth and using carbon paper, to ensure equal distribution of occlusal forces and absence of interferences on all the functional movements of the mandible. At the same session, the orthodontic archwires were removed, revealing the new smile. The denture was again examined in the 3‐ and 6‐month recalls. Both the condition of the intraoral tissues and the appearance and function of the denture were found excellent (Figure 14). The patient remains under regular follow‐up by the members of the team, at 3‐month intervals.

Figure 14(a) Intraoral view of the RPD. (b) Patient′s appearance with the denture in place.

**Figure:**
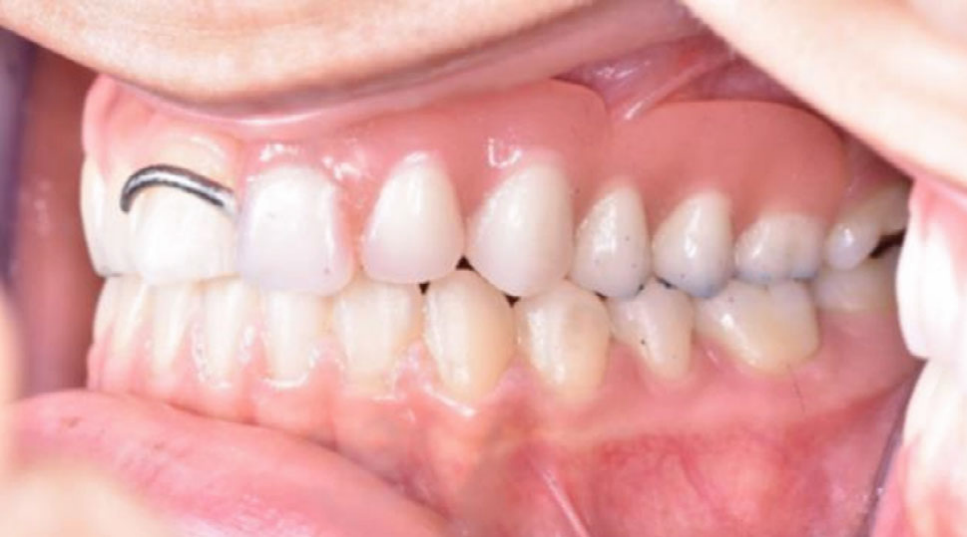
(a)

**Figure:**
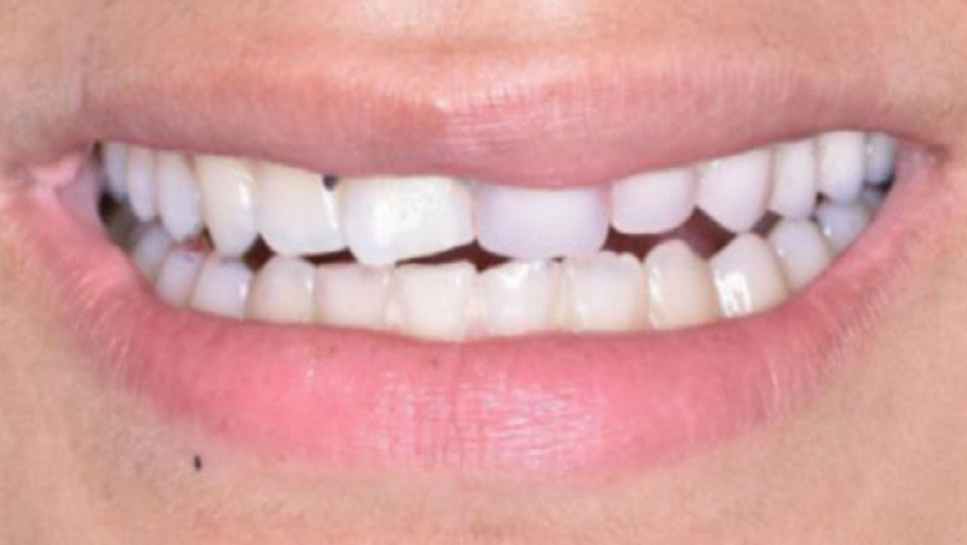
(b)

## 3. Discussion

Due to its unique clinical presentation, its early onset, and unavoidable progression, RO presents a major challenge for the clinicians called upon for it. Early diagnosis and targeted treatment by a coordinated multidisciplinary team are necessary for effective management [28, 29].

The pediatric dentist has an essential role in the early diagnosis of the disease and initiation of its management. The differential diagnosis is mainly based on the location of the impairment, as RO usually affects only specific segments and not the entire dentition [30]. In the present case, the diagnosis of RO was established through the combined evaluation of clinical, radiographic, and histological findings. The affected teeth exhibited hypoplastic, hypocalcified, and discolored crowns with soft consistency and abnormal morphology, localized in a single maxillary quadrant. The differential diagnosis included other developmental dental anomalies such as amelogenesis imperfecta and dentinogenesis imperfecta. However, these conditions were excluded based on the regional distribution of the lesions, the unaffected contralateral dentition, and the absence of hereditary or systemic findings. This diagnostic reasoning guided the clinical decisions and confirmed the diagnosis of RO.

The pedodontic follow‐up, installed early after the diagnosis, preserved the health of the impaired area, as well as the whole of the oral tissues. The regular contact with the patient and parents established and retained their cooperation and trust through the 12‐year treatment. The multidisciplinary team was thus able to organize the treatment strategy and apply the targeted interventions at the proper timing.

The management protocol for growing patients with multiple missing teeth involves systematic monitoring throughout their growth years, as well as timely prosthodontic and orthodontic interventions. These interventions aim to improve appearance and function, regulate the growth of the dentoalveolar complex, and create the best conditions for permanent restoration once the patient reaches adulthood.

For achieving the above goals, orthodontic interventions are frequently needed. In the current case, the orthodontic treatment was timely applied, short, and effective. It corrected the patient′s dental relationships, improved the occlusion by teeth movements in the three planes, and generated valuable prosthodontic space. The restoration of missing teeth and improvement of oral function are expected to promote the proper evolution and maturation of the stomatognathic system.

Two RPDs, constructed at successive steps of treatment, were the prosthodontic treatment of choice. Their aim was to alleviate the aesthetic and functional limitations arising from the early loss of teeth. Additionally, they provided a typical occlusal plane for the opposing teeth. Many reports on RO cases present the construction of prosthodontic replacements to serve for the years of growth [14, 28, 29, 31–34]. However, little information is offered about the design and construction of the appliances. When restoring little children, the prosthodontic protocols have to consider the special needs, background, scope, and anatomy. Although cases of restoring the missing or malformed teeth by fixed crowns of fixed partial dentures can be found in the literature, RPDs are preferable [8]. Removable prosthodontic appliances can be noninvasive, simple, and modifiable; thus, they serve well as temporary prostheses until adulthood. As scarce information can be found in the published reports, the present report is the first to describe the design, construction, and special problems associated with them.

The initiation of the prosthodontic treatment must be synchronized with the patient′s maturation, respond to his/her needs and demands, and secure his/her cooperation for the effective use of the removable prostheses. In the present case, while the first denture was being constructed, the patient′s determination for having a substitute for the missing teeth remained limited. She was reluctant to wear the first denture after it was constructed and abandoned the effort as soon as the anatomic changes in the mouth created problems in the fit of the appliance. It was only after some years, when she turned 15, that she confidently requested a new denture, and this time she was resolute in her decision.

When RPDs are constructed in children, the usual problems are the small area of support, the unfavorable morphology of the retaining teeth and the space limitations. Moreover, the continuous changes in the oral anatomy, as the patient grows, necessitate the close follow‐up of the restoration and frequent modifications. A major construction problem in the present case was the extremely restricted space that was available for the base and artificial teeth. This made the teeth set‐up a difficult task during the construction of the first denture; however, it was finally managed. The denture functioned satisfactorily, but only for a period of some months. The need for modifications came up relatively soon, as the supporting area changed. Consequently, the patient′s lack of compliance stalled the subsequent interventions that were planned. However, as the pediatric follow‐up continued uninterrupted, the patient remained in contact, and the team was able to confirm that her condition did not seriously deteriorate. Her treatment continued as soon as she was ready and willing to accept it. The second denture was more easily accommodated in the available space, and as the patient had considerably grown, the opposing teeth were aligned and the intervening Tooth #21 had been extracted.

The present case provides a long‐term (12 years) documentation of the multidisciplinary management of a patient with RO, from early childhood to adolescence. Unlike most previous reports that focus primarily on the diagnostic or radiographic features of the disease, our report highlights the restorative and orthodontic aspects of treatment, illustrating how prosthodontic management was adapted to the patient′s growth and evolving cooperation. The detailed description of the design, construction, and modification of the removable prostheses throughout the treatment offers useful insight for clinicians managing similar cases, serving as a reference for comprehensive long‐term rehabilitation of young patients with RO.

The patient remains under regular follow‐up. It is anticipated that the continuing growth, along with the provisional nature of the prosthodontic appliance, would necessitate further targeted interventions in the future, for the preservation of the outcome, and possibly the replacement of the RPD. Finally, when the patient reaches adulthood, a permanent treatment plan will have to be organized, for a more permanent restoration, preferably a fixed implant prosthesis on the edentulous spaces.

## 4. Conclusions

RO is a rare localized developmental anomaly causing severe dental disturbances. Individualized treatment with a multidisciplinary approach is of crucial importance. Coordinated and targeted interventions from pediatric, prosthodontic, and orthodontic specialists constitute the best treatment plan for each case. The present report adds useful information for the long‐term treatment and the successive treatment steps and underpins the necessity for compliance with the chosen therapy.

## Consent

Written informed consent was obtained from the patient′s parents for publication of this case report and any accompanying images.

## Disclosure

All authors have read and agreed to the published version of the manuscript.

## Conflicts of Interest

The authors declare no conflicts of interest.

## Author Contributions

Conceptualization: T.N. Methodology: E.K. Investigation: T.N., K.K., and F.A. Writing—original draft preparation: T.N. Writing—review and editing: E.K. Visualization: T.N., K.K., and K.P. Supervision: O‐E.K., K.A., and E.K. Project administration: E.K.

## Funding

No funding was received for this manuscript.

## Data Availability

All relevant data supporting the conclusions of this case report are included within the article. Additional information derived from patient records is not publicly available due to privacy restrictions.
